# Triazole analogues as potential pharmacological agents: a brief review

**DOI:** 10.1186/s43094-021-00241-3

**Published:** 2021-05-25

**Authors:** Sachin Kumar, Sukhbir Lal Khokra, Akash Yadav

**Affiliations:** Institute of Pharmaceutical Sciences, Kurukshetra University, Kurukshetra, Haryana 136119 India

**Keywords:** Analgesic, Anthelmintic, Anti-inflammatory, Antimicrobial, Triazole

## Abstract

**Background:**

A large number of studies have recently reported that, because of their significant biological and pharmacological properties, heterocyclic compounds and their derivatives have attracted a strong interest in medicinal chemistry. The triazole nucleus is one of the most important heterocycles which has a feature of natural products as well as medicinal agents. Heterocyclic nitrogen is abundantly present in most medicinal compounds. The derivatization of triazole ring is based on the phenomenon of bio-isosteres in which substituted the oxygen atom of oxadiazole nucleus with nitrogen triazole analogue.

**Main text:**

This review focuses on recent synthetic procedure of triazole moiety, which comprises of various pharmacological activities such as antimicrobial, anticonvulsant, anti-inflammatory, analgesic, antitubercular, anthelmintic, antioxidant, antimalarial, antiviral, etc..

**Conclusion:**

This review highlights the current status of triazole compounds as different multi-target pharmacological activities. From the literature survey, triazole is the most widely used compound in different potential activities.

## Background

In the field of research and the synthesis of new bioactive molecule, heterocyclic chemistry plays the most important role. Medicinal chemistry is a part of the medical and pharmaceutical sciences, is concerned with the development and design, and credits the significant biologically active drug molecule. The most active biological activities have been shown among these heterocyclic molecules containing nitrogen and oxygen. Many different compounds have been prepared and exhibit different types of useful pharmacological activity [1].

To investigate a new agent is one of the most difficult tasks for the medicinal chemist. Synthesis of heterocyclic systems consisting high nitrogen has been rising over the past decade owing to their usefulness in different applications such as propellants, explosives, pyrotechnics, and especially chemotherapy. In recent years, considerable attention has been received by the chemistry of triazoles and their fused heterocyclic derivatives because of their synthetic and effective biological importance [2].

Azolic derivatives such as thiazole, triazole, oxadiazole, and thiadiazole are pharmacologically active compounds and, due to their effective use in medicinal chemistry, have been intensely studied for various biological activities [3].

## Main text

### Triazole

**Fig. 1:**
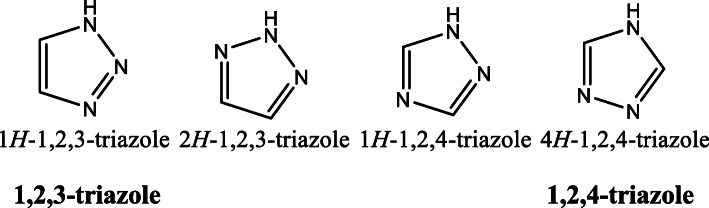
Different isomeric forms of triazole

Triazole is a five-member heterocyclic ring containing two carbon and three nitrogen atoms with molecular formula C_2_H_3_N_3_ [4]. And it is found in two isomeric forms, 1,2,3-triazole and 1,2,4-triazole, which are also known as pyrrodiazole. (Fig. 1).

**Fig. 2:**
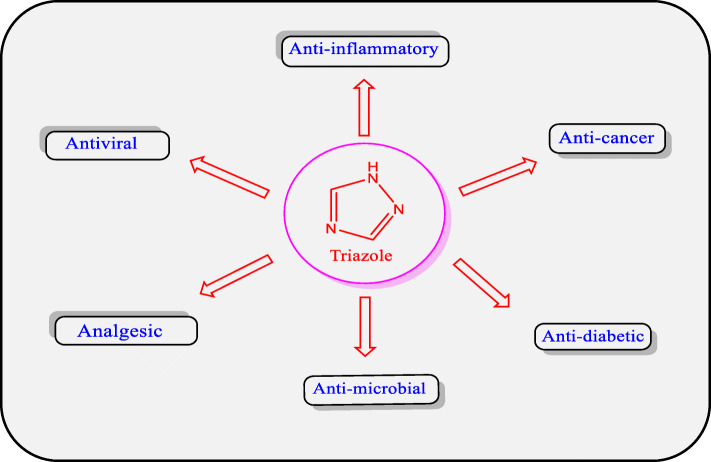
Significant biological activities of triazole derivatives

Triazoles are white-to-pale yellow crystals with a weak odour, soluble in water and alcohol at a melting point of 120 °C and 260 °C [5]. In medicinal chemistry, five-member heterocyclic nitrogen-containing compounds such as triazole are of great importance due to their wide range of biological applications such as anticonvulsant [6, 7], antimicrobial [8, 9], antiviral [10, 11], antitubercular [12], antidiabetic [13], anti-inflammatory [14, 15], anti-proliferative [16–18], antioxidant [19], anti-urease [19], and antimalarial activities [20, 21]. (Fig. 2).

### Synthetic approaches of triazoles

The article gives a brief review of the synthetic procedure and characterization of triazole and its pharmacological activity.

**Scheme 1:**
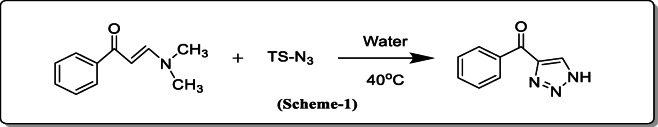
Synthesis of 4-acyl-NH-1,2,3-triazole

*Lu yang et al.* reported 4-acyl-NH-1,2,3-triazole synthesis by the use of water-mediated cycloaddition reactions of enaminone and tosilazide, requiring both a mild condition (40 °C) and practical scalability, when using the water as sole medium without any catalyst (Scheme 1) [22].

**Scheme 2:**
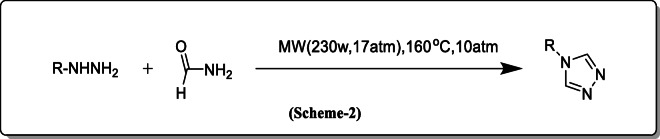
Synthesis with substitution of 1,2,4 triazole from hydrazine and formamide

*Shelke et al.* had synthesized and found that, in the absence of a catalyst, the substitution of 1,2,4 triazole from hydrazine and formamide under microwave irradiation and this reaction effectively indicates excellent functional group tolerance (Scheme 2) [23].

**Scheme 3:**
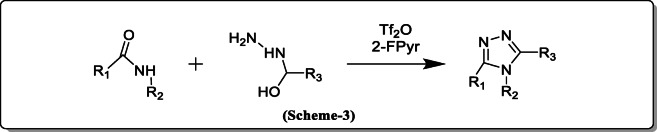
Synthesis of 3,4,5-Trisubstituted 1,2,4-triazole from 2 amides and hydrazides

*Bechara et al.* reported the synthesis of 3,4,5-Trisubstituted 1,2,4-triazole from 2^o^ amides and hydrazides by triflic anhydride activation followed by the microwave cyclodehydrationto be1,2,4-Triazole moiety is a useful leading group of Ru-catalyzed C-H arylation (Scheme 3) [24].

**Scheme 4:**
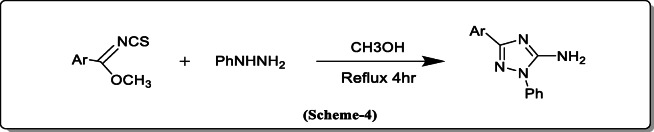
Synthesis of a substituted triazole by one pot cyanoimidation of aldehydes

*Yin et al.* synthesized a substituted triazole by one pot cyanoimidation of aldehydes where cyanamide as nitrogen source and the NBS as an oxidant in high yield without any catalyst. The substituted product N-cyanobenzimidate may also be subjected to a cyclization reaction to produce a high yield of 1,2,4-triazole derivative (Scheme 4) [25].

**Scheme 5:**
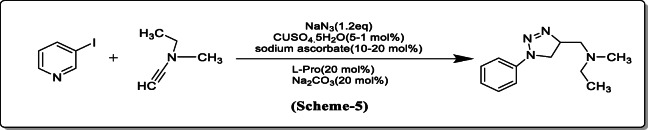
1,4 Disubstituted 1,2,3 triazoles from azides

*Faldiman et al.* reported 1,4 disubstituted 1,2,3 triazoles from azides. These are obtained with excellent yield from aromatic and aliphatic halides that are easily available without formation of potentially unstable organic azide intermediates (Scheme 5) [26].

**Scheme 6:**
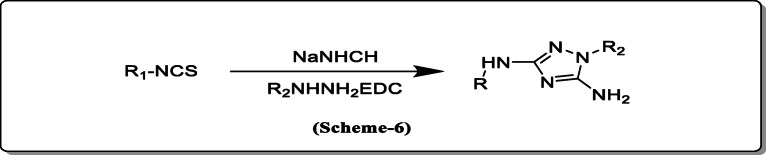
Novel substituted 3,5-diamine-1,2,4-triazole

*Liu et al.* reported a novel substituted 3,5-diamine-1,2,4-triazole from isothiocynate and mono-substituted hydrazines and sodium hydrogen cyanamide (Scheme 6) [27].

**Scheme 7:**
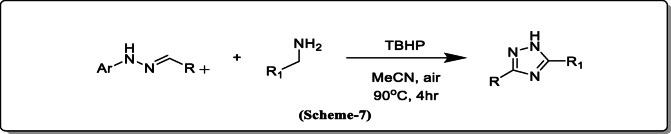
Metal-free synthesis of 1,3,5-trisubstituted-1,2,4-triazoles in the presence of iodine as catalyst

*Zhengkaichen et al.* reported a metal-free synthesis of 1,3,5-trisubstituted-1,2,4-triazoles in the presence of iodine as catalyst (Scheme 7). And it can be synthesized from hydrazones and aliphatic amines under oxidative conditions via a cascade C–H functionalization, double C–N bond formation, and oxidative aromatization [28].

### Pharmacological activities of triazole derivatives

This article presented discusses a brief description of the various triazole activities, and the recent studies have showed the wide range of pharmacological activities available for triazole derivatives which may be divided into the following categories:

#### Antimicrobial activity

*Fabrice et al.* synthesized a novel series of 1,2,4-triazole-indole hybrids and evaluated their antifungal activity. All the synthesized hybrids were characterized by IR, NMR, and mass and elemental spectroscopy. The compound (2-(2,4-Dichlorophenyl)-3-(1H-indol-1-yl)-1-(1,2,4-1H-triazol-1-yl) propan-2-ol **1a** exhibited the excellent activity against *Candida,* particularly against low fluconazole susceptible species. Result showed that this compound exhibited high activity as compared with fluconazole and similar to voriconazole against *C. glabrata*, *C. krusei,* and *C. albicans* [29].

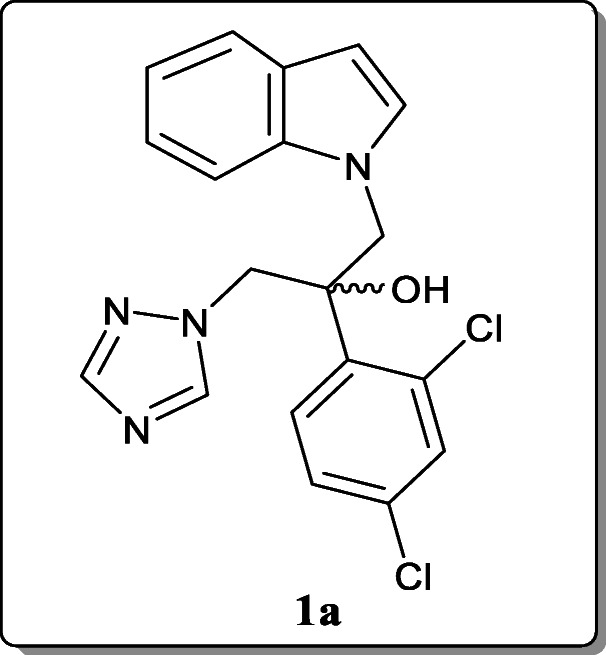



*Wujec et al.* synthesized the ten compounds which contain the manic base-1,2,4 triazole. The broth microdilution technique was used against Gram-positive and Gram-negative bacteria to evaluate antimicrobial activity of these compounds. The phenyl ring present in the 4-position of piperazine appears essential for antibacterial action. Compound **2a** showed the potent activity with MIC value 30 μg/mL against *M. luteus* and 60 μg/mL against three different bacterial strains (*B. subtilis, S. aureus,* and *S. epidermidis*) [30].

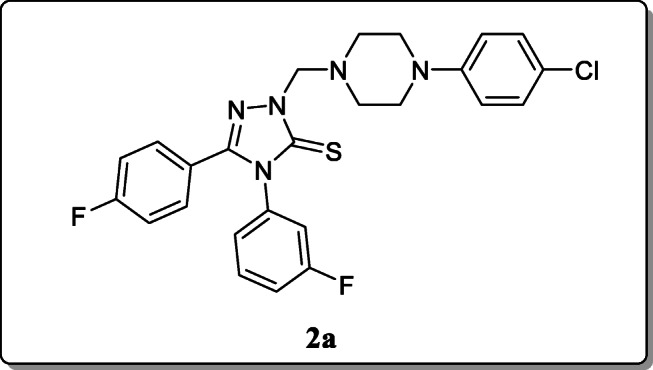



*Lipeeva et al.* synthesized and investigated a novel series of 1,2,3-triazole-substituted coumarins and tested their *in vitro* antimicrobial activity against four different bacterial strains. Result showed that compounds **3a**, **3b,** and **3c** showed potent activity against *S. aureus* strains with MIC values ranging between 0.16 and 0.41 μg/mL as compared with the reference drug ceftriaxone and streptomycin. The structure activity relationship of compound (carboxamidotriazolylbenzoic acid) substitution at position C-6 of coumarin core displayed promising activity towards *A. viscosus* as compared with compound **3b**. The compound **3b** with triazolylbenzoic acid substitute in the C-7 position exhibited highest activity towards the bacterial strains of *S. aureus* “Viotko”, and compound **3c** with the substitution of 3-ethynylcoumarin with methyl-anthranilate exhibited remarkable antibacterial activity against the strains of *S. aureus* [31]. 
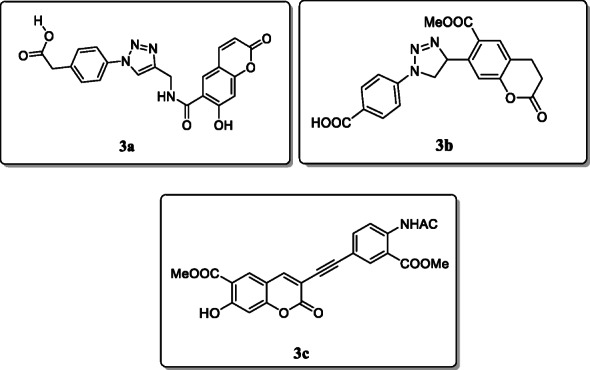


*Tang et al.* synthesized the triazolyl-pterostilbene derivatives, and their antimicrobial activity was evaluated. Among all these compounds, compound **4a** showed the most potent antimicrobial activity with MIC values of 1.2–2.4 μg/mL and MBC values of 19.5–39 μg/mL. On the other hand, structural activity analysis showed introduction of the phenyl group as a spacer on compound **4a** exhibited significant antimicrobial activity [32]. 
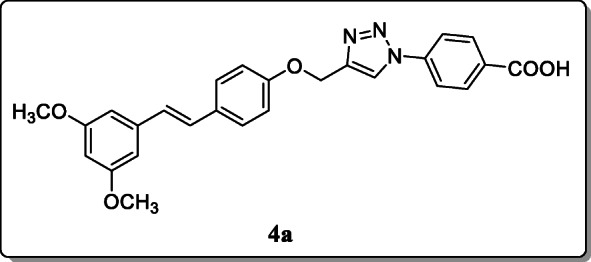


*Tingjunhong Ni et al.* synthesized twenty-seven triazole derivatives containing alkynyl side chains, and their antifungal activity towards *Cryptococcus* and *Candida* species were evaluated as compared with reference drugs. The results showed that the compounds **5a** and **5b** demonstrated *in vitro* activity towards all fungi with MIC_80_ values in range between 0.0156 and 0.5 μg/mL, higher than ravuconazole and fluconazole. Structural relationships showed the introduction of fluoro, chloro, and cyano groups at p-position of phenyl alkynyl or pyridinyl alkynyl side chain enhances their antifungal activity [33]. 
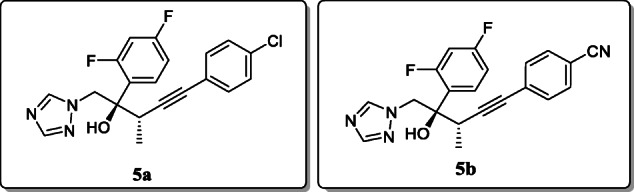


*Yang et al.* synthesized the derivatives of quinazoline (E)-2-(4-(1H-1,2,4-triazol1-yl) as an antimicrobial agent. Among these compounds, *in vitro* antimicrobial activity was evaluated against three phytophatogenic bacteria (*Xac*, *Xoo,* and *Rs*) as compared with the reference bismerthiazole (BMT) drug. Among them, compounds **6a**, **6b**, and **6c** showed better antibacterial activity against pathogen *Xac* and its EC_50_ values are 53.2, 67.7, and 70.7 μg/mL. And the antifungal activity also evaluated against the three phytopathogenic fungi. Result revealed that the compounds **6c**, **6d**, **6e**, and **6f** showed the modest inhibition activities with EC_50_ values 45.7 ± 1.8, 40.7 ± 2.1, 43.6 ± 1.7, and 43.1 ± 2.1 respectively against *S. sclerotiorum* with the reference of Hymexazol at 50 μg/mL, having > 40% inhibition rate [34] where value of R in 6a. R = C(CH3)3C6H4, 6b. R = 2,6 -Cl2C6H3, 6c. R = 4 -FC6H4, 6d. R = CH3, 6e. R = (CH2)2CH3, 6f. = C6H5. 
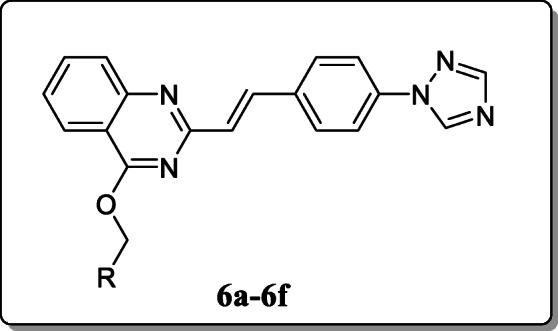


*Rezki et al.* reported and investigated a novel series of 2,5-disubstituted thiadiazole clubbed 1,2,4-triazole as a potential antimicrobial agent. All derivatives were characterized by IR, ^1^H-NMR, ^13^C-NMR, MS, and elemental analysis. *In vitro* inhibitory growth activities of three Gram-positive (+) bacteria, three Gram-negative (-) fungi, and three strains of normal pathogenic microorganism strains were tested of all these compounds. SAR studies revealed the presence of phenyl or alkyl substitution at N-4 has enhanced their antimicrobial activity towards strains of bacteria and fungi with MIC values of 8–16 μg/mL, where ciprofloxacin and fluconazole are the reference drugs (Table 1). Compounds **7a–7c** were found to be the most potent antimicrobial agent [35]. 
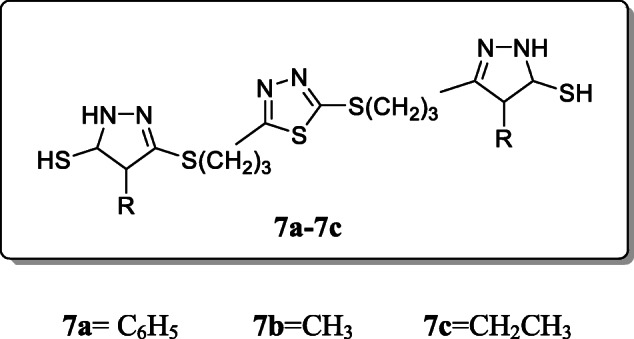

**Table 1:** Antimicrobial activity expressed as MIC (μg/mL)

Comp.	Gram positive			Gram negative			Fungi		
	*Sp*	*Bs*	*Sa*	*Pa*	*Ec*	*Kp*	*Af*	*Ca*	*Gc*
7a	8	8	16	16	16	16	8	8	16
7b	16	31.5	16	31.5	31.5	8	16	16	31.5
7c	16	16	8	31.5	16	16	16	31.5	16
Ciprofloxacin	< 5	< 1	< 5	< 5	< 1	< 1	-	-	-
Fluconazole	-	-	-	-	-	-	< 1	< 1	< 1

*Tijenonkol et al.* reported the 3-[1(2H)-phthalazinone-2yl(substituted)-4-aryl-1,2,4-triazole-5-thione derivatives and evaluated their antibacterial activity and screened them against Gram (+) & Gram (-) bacterial strains and fungal strains by using the broth microdilution method. Result revealed that the compounds **8a–8e** exhibited the antibacterial activity is 25% against *B. subtilis*. And the antifungal activity of compound **8c** was found to be 25% against *C. albicans*. The MIC value of compound **8e** towards *C. albicans* and *C. parapsilosis* was 64 μg/mL & 32 μg/mL, and compound **8d** was active towards *C. parapsilosis* with MIC value 32 μg/mL (Table 2) [36]. 
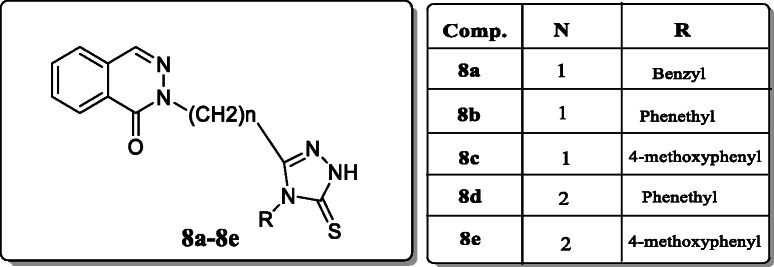

**Table 2:** Minimum inhibition of concentration compound (μg/mL)

Comp.	*S. aureus*	*B. subtilis*	*E. coli*	*P. aeruginosa*	*C. albicans*	*C. parapsilosis*
**8a**	512	32*	256	256	128	64
**8b**	256	256	128	256	32*	32*
**8c**	512	32*	256	256	512	512
**8d**	512	32*	256	256	64*	32*
**8e**	512	64	256	256	64	32*

*Turan-Zitouni et al*. synthesized 4-phenyl-cyclohexyl-5-(1-phenoxyethyl)-3-[N-(2-thiazolyl)acetamido] thio-4H-1,2,4-triazole analogues and tested their antimicrobial activity. Among these synthesized compounds, only compound **9a** showed excellent antifungal activity [37]. 
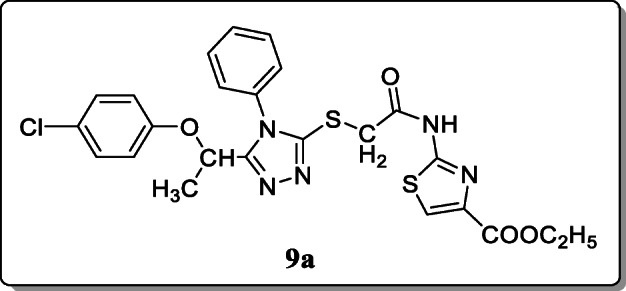


*Hussain et al.* synthesized eleven 1,4-disubstituted-1,2,3-triazole derivatives for antibacterial activity. All the synthesized derivatives were characterized spectroscopically, and their activities were evaluated. And the preliminary results of the synthesized derivatives showed the high inhibitory effects compared with the control ciprofloxacin. Result showed that the compounds **10a** and **10b** were found to be potent (MIC: 5 μg/mL, MIC: 10 μg/mL respectively) antibacterials against various strains of bacteria. And the docking studies showed that the most potent is compound **10a,** exhibiting high binding energy and inhibition constant [38]. 
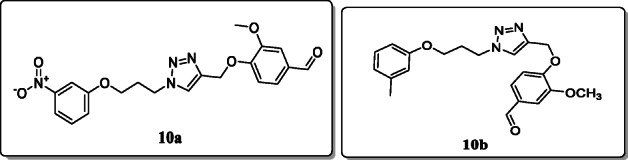


*Han et al.* reported a new series of triazole derivatives containing different ester skeletons and evaluated as antifungal agents. The antifungal activity was investigated by utilizing the microdilution broth method. In all the synthesized compounds, compounds **11a** and **11b** showed the most significant activity against four important fungal pathogens (MIC_80_ = 2–8 μg/mL). Molecular docking studied revealed the target compounds interact with CYP51 mostly by Van der Waals and hydrophobic interactions [39].

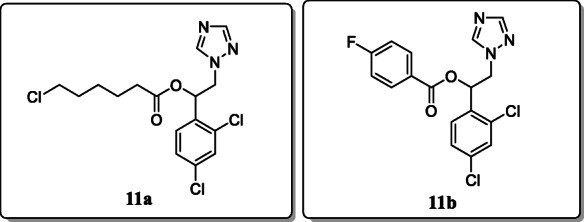



*Al-blewi et al.* synthesized a novel series 1,4-disubstituted-1,2,3-triazole-sulfonamide hybrids and evaluated for their antimicrobial activity. All the synthesized hybrids were verified by mean of spectroscopic analysis. From the result, only compound **12a** showed the most significant activity with MIC value range between 32 and 64 μg/mL as compared with the standard drug [40]. 
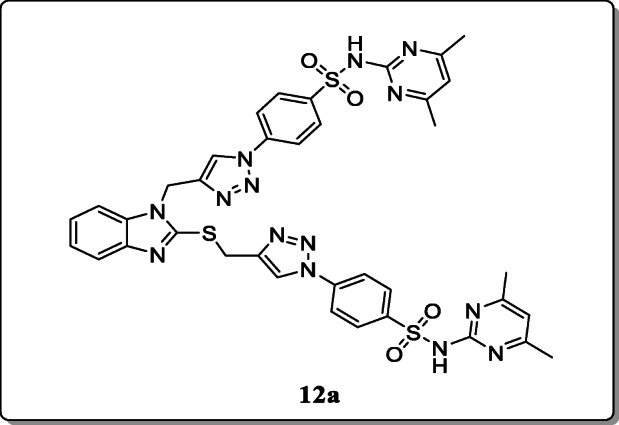


#### Antitubercular activity

*Ramprasad et al.* reported nineteen derivatives of quinoline-triazole hybrids and screened their antitubercular activity against *Mycobacterium bovis.* Result revealed that two derivatives, **13a** and **13b**, showed the potent antitubercular activity with MIC values 31.5 μm and 34.8 μm. SAR studies revealed that these compounds are essential for their activity due to n-octyl and 3-fluorophenyl groups presented on 1,2,3-triazole ring [41]. 
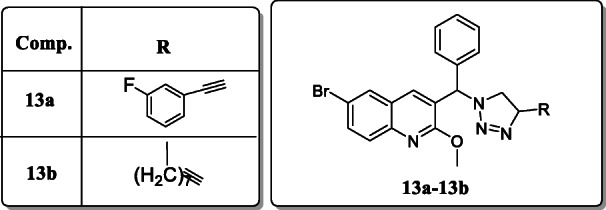


A novel series of triazole–imidazo[2,1-b][1,3,4]thiadiazole hybrids and evaluated their antimycobacterial activity against *Mycobacterium tuberculosis H37Rv* strain reported by *Rampprasad et al*.. From the result, two derivatives **14a** and **14b** demonstrated potent growth inhibition towards the bacterial strain with significant MIC value 3.125 μg/mL. Substitution of the ethyl benzyl group on 1,2,3-triazole ring enhances the inhibition activity [42]. 
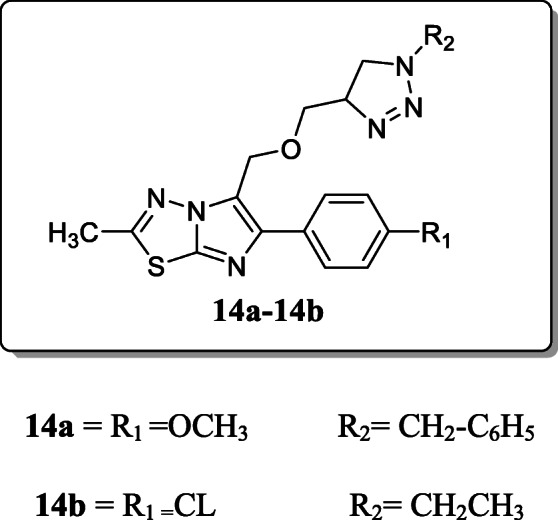


*Raju et al.* synthesized 1H-pyrrolo[2,3-d]pyrimidine-1,2,3-triazole derivatives for *in vitro* antimycobacterial activity against *Mycobacterium tuberculosis* H37Rv strain. All synthesized hybrids exhibited significant antitubercular activity. Among these series, compounds **15a** and **15b** showed the remarkable MIC value 0.78 μg/mL. The molecular docking results to the exhibition of high Moldock score of these compounds. SAR studies showed that the triazole ring substituted with heteroaryl compound containing highly electronegative atoms also enhance their activity [43]. 
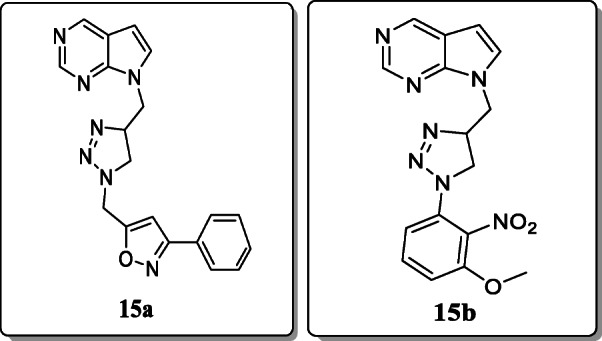


*Patela et al.* reported a series of N-Mannich base of 1,2,4-triazole derivatives. All the synthesized derivatives were characterized by spectral and elemental analysis and were screened for *in vitro* antitubercular activity against *M. tuberculosis*. From the result, in the primary screening, compound **16a** revealed the remarkable activity (MIC = 6.25 μM) against *M. tuberculosis.* The computational studies showed a high affinity towards the active enzyme site for that Mannich derivative **16a** that provides a strong platform for new structure-based design efforts [44]. 
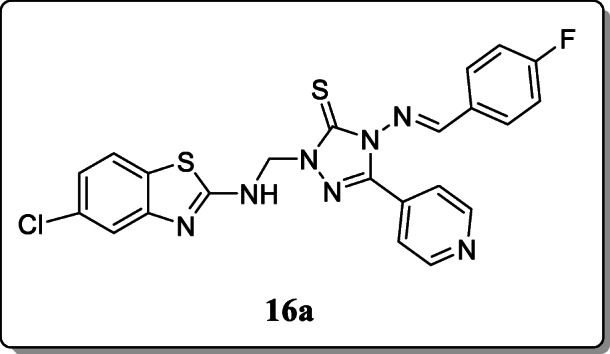


*Ali et al.* reported and investigated seventeen new 1,2,3-triazole derivatives against *Mycobacterium tuberculosis* H37Ra (ATCC 25177 strain)*.* The synthesized compounds were characterized by thin-layer chromatography (TLC), ^1^H NMR, ^13^C NMR, FT-IR, and mass spectrometry. Among the tested series, compound **17a** substituted with the fluoro group at second position on the phenyl ring of the triazole derivatives demonstrated higher anti-mycobacterial activity with MIC = 0.78 μg/mL as compared with the first-line antitubercular drug ethambutol (MIC = 2.00 μg/mL). However, the compound **17b** with the ester group also showed significant activity (MIC = 1.56 μg/mL), in contrast with its antimicrobial activity [45]. 
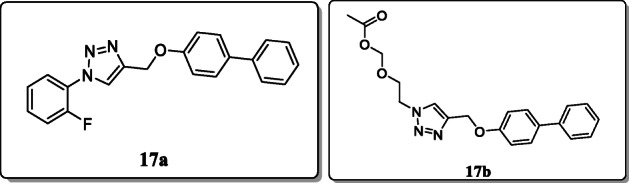


#### Anthelmintic activity

*Kharb et al.* investigated fifteen novel imidazole-containing triazole derivatives and screened their anthelmintic activity towards *Pheretimaposthuma* at concentrations of 0.150% and 0.300% w/v respectively as compared with the albendazole as positive control. Result revealed that, the compound **18a** displayed significant anthelmintic activity as compared with the reference drug [46]. 
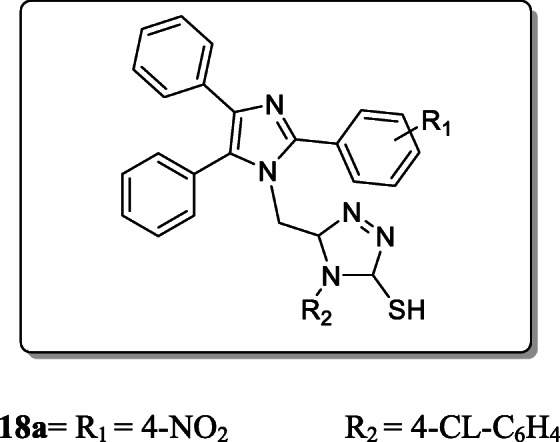


*Gupta et al.* reported five derivatives and evaluated for their anthelmintic activity against *P. posthuma*. From the result, compound **19a** showed the potent vermicidal activity, and it exhibited the maximum paralysis time and 37.33 min of death time at 20 mg/mL concentration [47, 48]. 
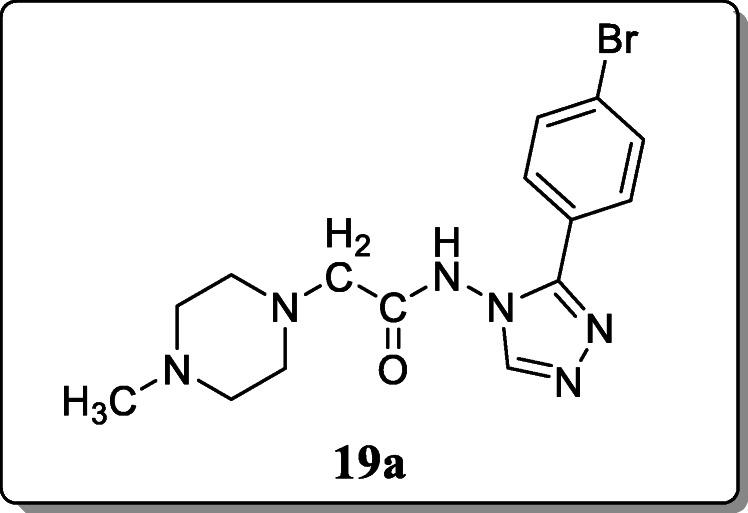


*Satyendra et al.* synthesized novel di-chloro substituted benzoxazole-triazolo-thione derivatives, and their anthelmintic activities were evaluated. Among them, the compound **20a** exhibited the potent anthelmintic activity against *P. posthuma* as compared with the reference albendazole at 1% concentration [49]. 
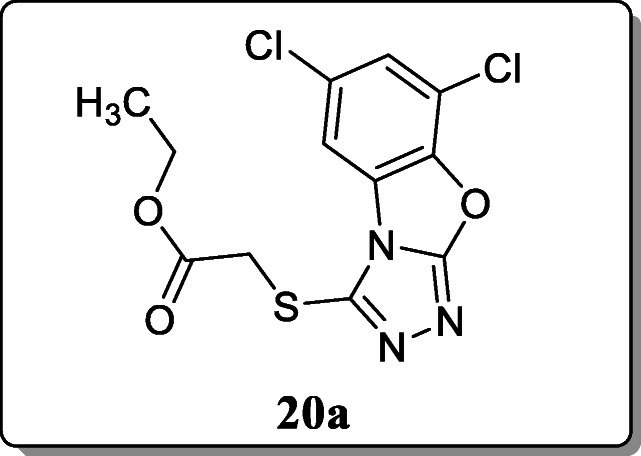


#### Anticonvulsant activity

*Verma et al.* reported a series of novel 4,5-disubstituted-2,4-dihydro-3H-1,2,4-triazole-3-thione derivatives for anticonvulsant activity. Anticonvulsant activity of compound wastes by maximal electroshock (MES), subcutaneous pentylenetetrazol (scPTZ) test in mice and rat and neurotoxicity screened at 30, 100, and 300 mg/kg dose and was suspended in 30% PEG 400 by an oral route to the mice. Among all these compounds, only compound **21a** exhibited significant anticonvulsant activity at 300 mg/kg at a 4-h duration [50]. 
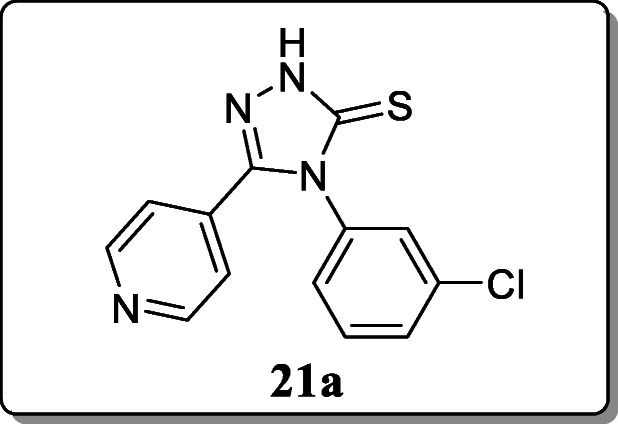


*Wang et al.* reported a novel series of triazole-containing 7-phenyl-4,5,6,7-tetrahydrothieno[3,2-b]-pyridine derivatives and screened their anticonvulsant activity. From the result, compound **22a** exhibited the potent anticonvulsant activity. Out of the therapeutic index (PI) values, compound **22a** displayed better safety profile than carbamazepine and ethosuximide [51]. 
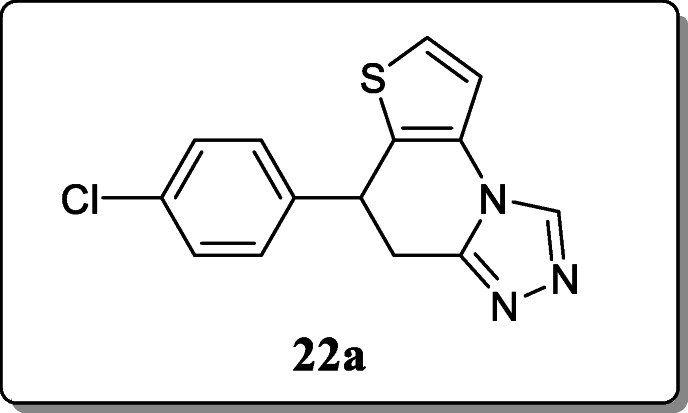


*Zhang et al.* synthesized a new series 3,4-dihydroisoquinolin containing 1,2,3-triazole compounds and investigated their anti-epileptic activity by using MES (maximal electroshock) and PTZ (pentylenetetrazole)-induced seizure test. Among the synthesized compound, only compound **23a** showed excellent anti-epileptic activity with ED_50_ value 48.19 mg/kg. It was found to be more active than valproate but less active than carbamazepine [52]. 
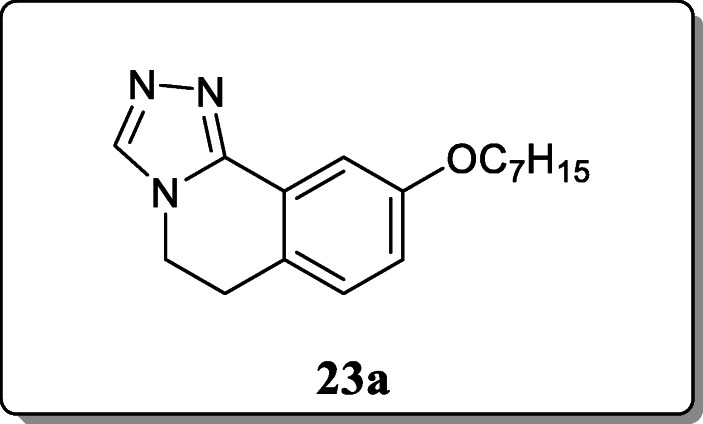


*Mahdavi et al.* synthesized a novel series of 3-Amino-5-[4-chloro-2-phenoxyphenyl]-4H-1,2,4-triazoles derivatives and evaluated for their anti-epileptic activity. Result showed that the only compound **24a** was found to have the most significant activity as compared with the reference drug [53].

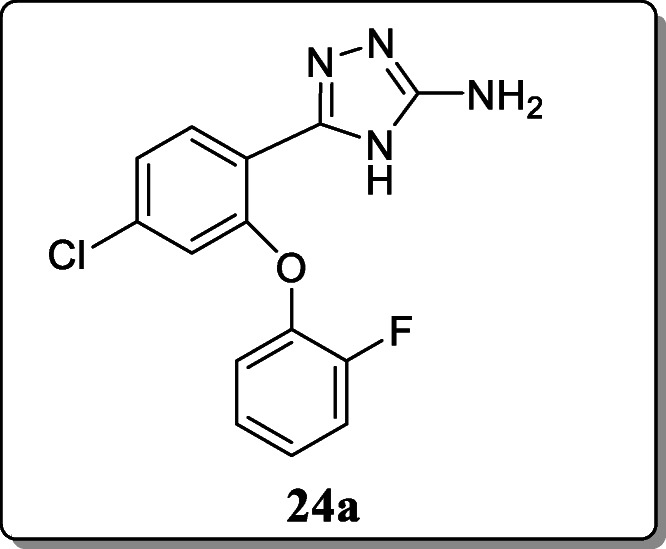



*Song et al.* reported a new series of 4-(2-(alkylthio)benzo[d]oxazol-5-yl)-2,4-dihydro-3H-1,2,4-triazol-3-one derivatives and evaluated their anticonvulsant activity. Two seizure models, the maximal electroshock seizure (MES) and subcutaneous pentylenetetrazole (scPTZ), were used for the anticonvulsant activity. From the result, only compound **25a** was found to be most significant compound with ED_50_ values of 23.7 and 18.9 mg/kg, respectively. Furthermore, the seizure-preventing action of compound **25a** the anticonvulsant activity confirmed by the 3-MP- and BIC-induced seizure models [54]. 
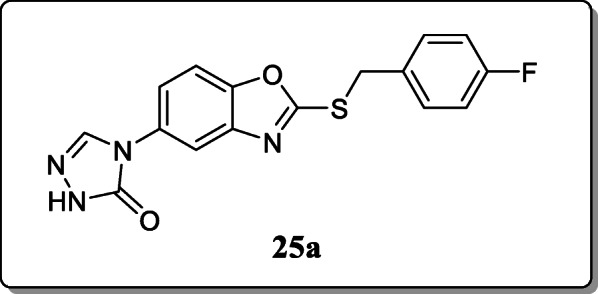


*Dehestani et al.* synthesized twelve phenacyl triazole hydrazone derivatives and screened their in vivo anticonvulsant activity by using the MES and PTZ seizure models. All synthesized derivatives are characterized by spectral analysis. Among the series, compound **26a** revealed the significant activity in both models. The computational studies of compound **26a** with different targets hypothesize that the compound acts mainly as a GABA_A_ receptor [55]. 
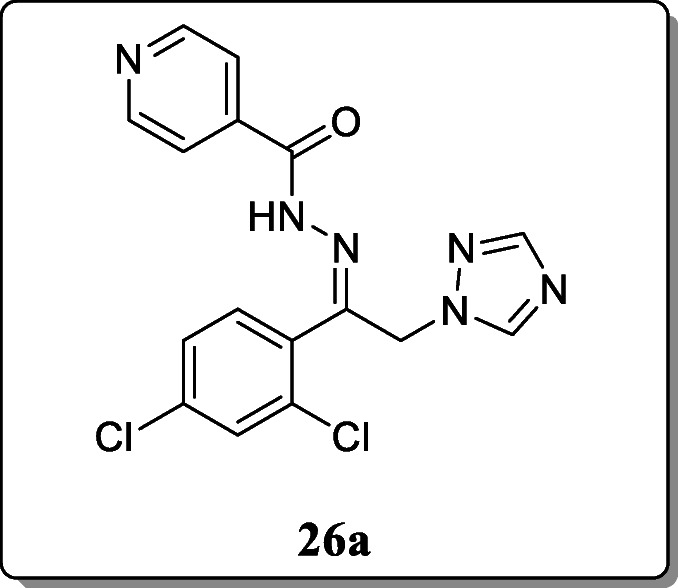


*Deng et al.* reported a novel series of 7-phenyl-6,7-dihydro -[1, 2, 4] triazolo[1,5-a]pyrimidine-5(4H)-ones derivatives and evaluated their anticonvulsant activity. Most of the synthesized derivatives showed the significant activity in the MES model. Out of all derivatives, compound **27a** displayed the most potent anticonvulsant activity with ED_50_ value 19.7 mg/kg [56]. 
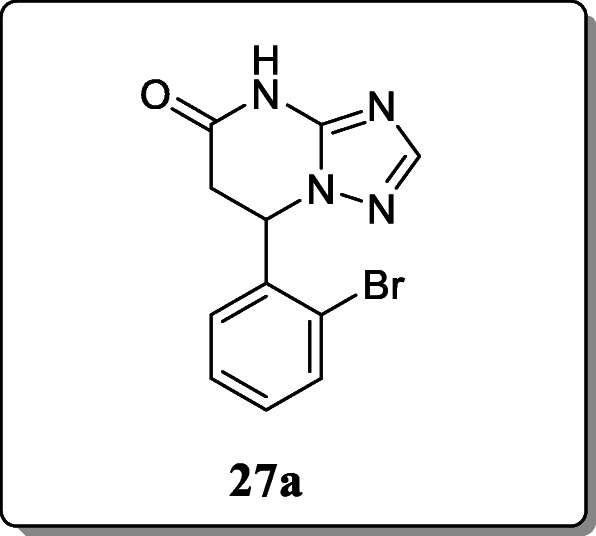


*Siddiqui et al.* synthesized various triazoles containing thiazole derivatives. The two most active compounds 47 and 48 were tested in the Phase II anticonvulsant study for their anticonvulsant activity (ED_50_) and neurotoxicity (TD_50_). And anticonvulsant action was carried out by two methods mostly using the electroshock (MES) and chemo shock (scPTZ) models. From the result, compounds **28a** and **28b** exhibited the potent anticonvulsant activity [57]. 
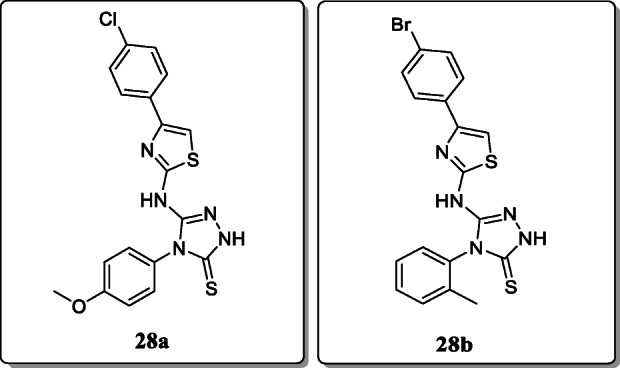


*Zheng et al.* synthesized a novel series of 4-(4-substitutedphenyl)-3-methyl-1H-1,2,4-triazole-5(4H)-one derivatives and evaluated their anticonvulsant activity. All the synthesized derivatives were characterized by NMR, IR, and mass spectroscopy. Among the series, compound **29a** was found to have the most promising activity with ED_50_ value of 25.5 mg/kg [58]. 
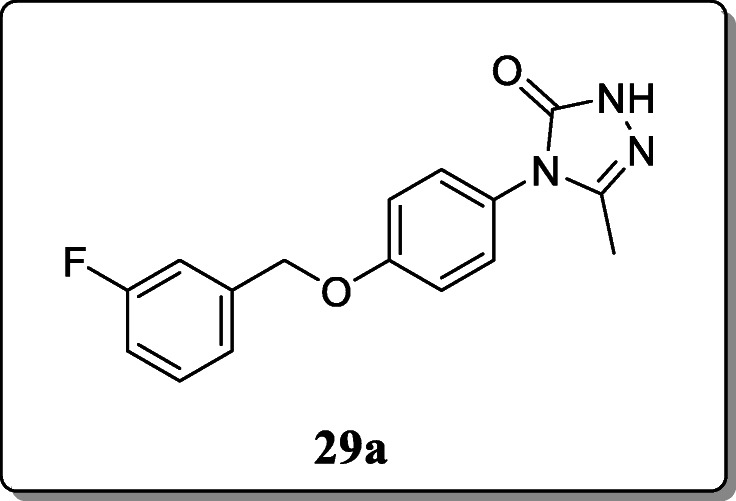


#### Analgesic and anti-inflammatory activity

*Tariq et al.* reported a novel class of N-[3-(substituted-4H-1,2,4-triazol-4-yl)]benzo-(d)] thiazol-2-amine derivatives and evaluated for their *in vivo* anti-inflammatory activity. From the result, only compound **30a** displayed the most potent *in vivo* anti-inflammatory [59]. 
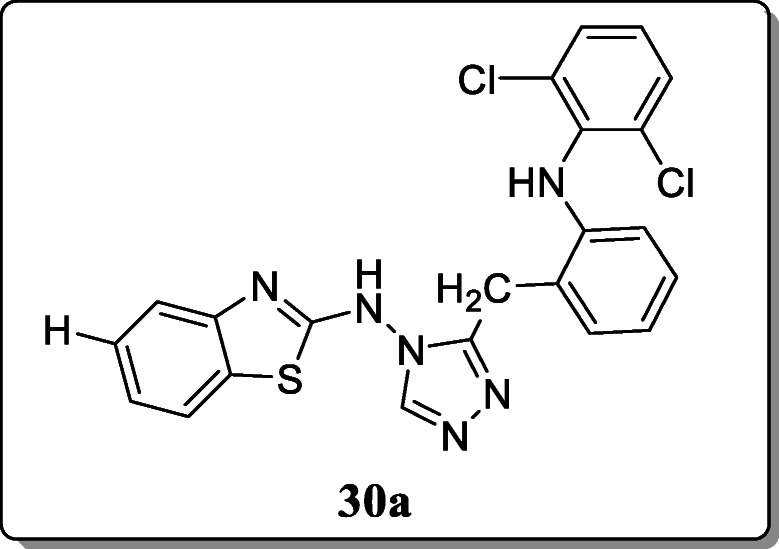


*Ahirwar et al.* reported a new series of substituted benzyl groups via thio-linkage and potential merged pharmacophore containing 1,2,4-triazoles and evaluated their analgesic and anti-inflammatory activities in mice and rats, respectively. Among all these derivatives, 3-(5-(2,4-dimethylbenzylthio)-4H-1,2,4-triazol-3-yl) pyridine **31a** showed excellent anti-inflammatory activity, and 3-(5-(4-nitrobenzylthio)-4H-1,2,4-triazol-3-yl) pyridine **31b** showed significant analgesic activity [60]. 
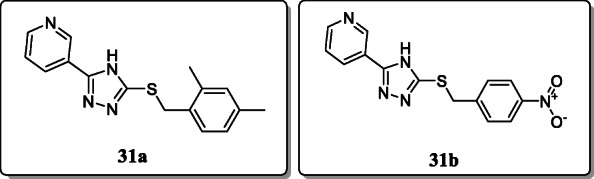


*Khan et al.* reported and investigated a new series of five membered heterocyclic derivative containing three hetero atoms for their *in vivo* anti-inflammatory activity. Among all the synthesized compounds, only compound **32a** showed the potent anti-inflammatory activity with 56.49% inhibition and the rest of the compounds showed moderate activity. And analgesic activity of all derivatives ranges between 27.50 and 65.24% as compared with the controlled drug [61]. 
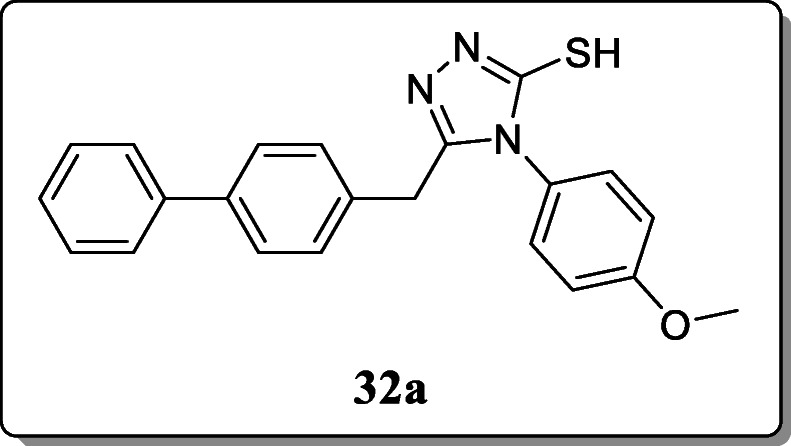


*Zhang et al.* synthesized a novel series of pyrimidine derivatives containing triazole and investigate their anti-inflammatory activity. Result revealed that the compound **33a** showed the significant anti-inflammatory activity with an inhibition rate of 49.26%. And other western blotting showed the dose-dependent NF-SB (p65) activation and MAPK (ERK) and p38-phosphorylation in dose response and concentration dependent manner is inhibited by this compound extract [62]. 
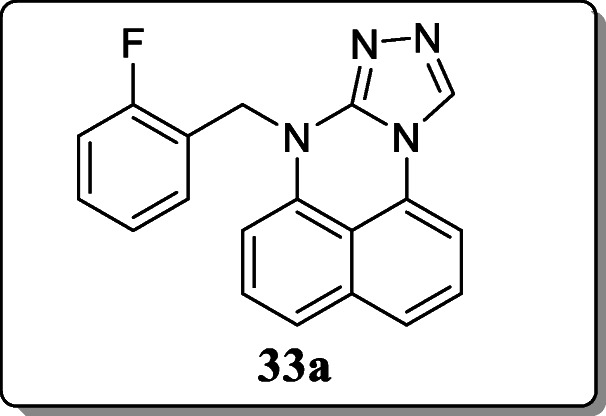


*Sarigol et al.* synthesized some thiazolo[3,2-b]-1,2,4-triazole-6(5H)-one derivatives and screened for their *in-vivo* analgesic and anti-inflammatory activity. Out of all derivatives, compound **34a** had the most selective COX-2 inhibition of all tested compounds and significant analgesic and anti-inflammatory activity [63]. 
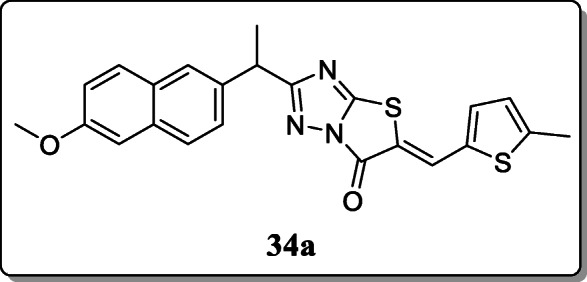


*Almasirad et al.* reported a new series of novel 1,2,4-triazole derivatives and screened their analgesic activity. And analgesic activity was evaluated by formalin-induced nociception test. Result revealed that the compound **35a** with the inhibition rate 49.38% in early phase and 79.62% in late phase showed the potent analgesic activity [64]. 
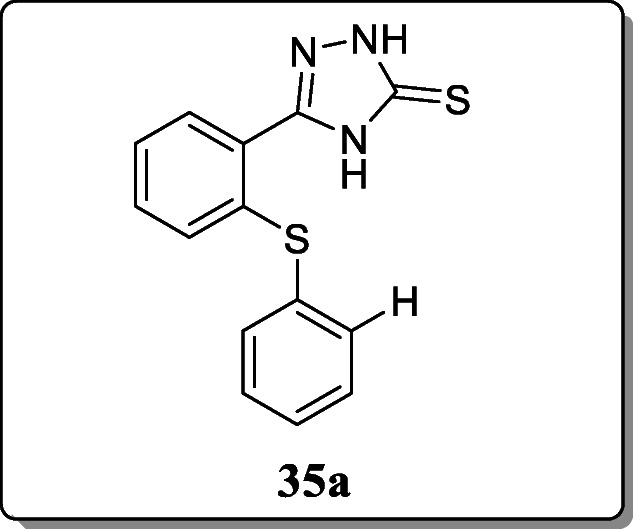


*Haider et al.* synthesized a series of 1,2,3-triazole-based benzoxazolinone and screened for their COX-2 inhibitory activity. From the result, compound **36a** exhibited the potent selective COX-2 inhibition (COX-1 IC_50_ = 174.72 μM; COX-2 IC_50_ = 2.4 μM) as compared with celecoxib. And the selective index of this compound shows the selective nature of the compound towards COX-2 inhibition. Compound **36b** also exhibited the significant antinociceptive activity [65]. 
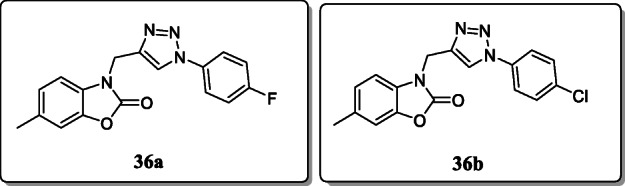


*Syed Shaf et al.* reported a novel series of bis-hetero cycles containing 2-mercapto benzothiazole-based 1,2,3-triazole and screened their anti-inflammatory activity. From the result, compound **37a** display the significant selective COX-2 inhibition activity as compared with the standard drug and compound **37b** also exhibited the comparable analgesic activity [66]. 
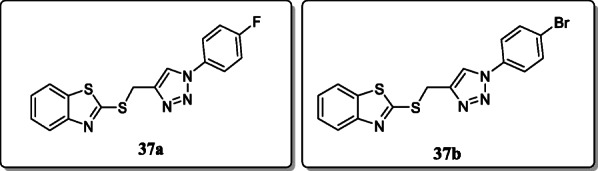


*Gamal El-Din A.A. et al.* described a novel series of 1-[4-(Aminosulfonyl)phenyl]-1H-1,2,4-triazole derivatives. All the synthesized derivatives were confirmed by different spectroscopic method. Among the tested series, compounds **38a**, **38b**, **38c**, and **38d** exhibited potent anti-inflammatory activity. SAR studies demonstrated that the substitution of 4-methoxyphenyl **(38a)**, 4-methylphenyl **(38b)**, 4-acetylphenyl **(38c)**, and 3,4-dimethoxyphenyl **(38d)** groups also increase anti-inflammatory activity as compared with the other derivatives [67].

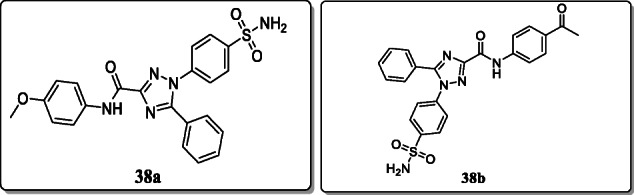

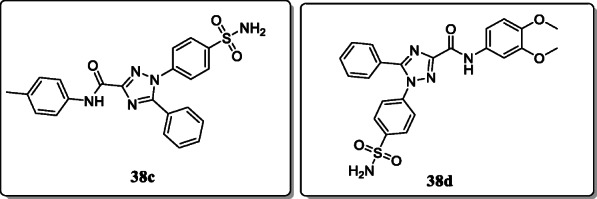





*Tozkoparan et al.* synthesized a novel series of 5-aryl-3-alkylthio-1,2,4-triazole derivatives and screened their anti-inflammatory activity. All the synthesized derivatives were characterized by spectral and elemental analysis. Among the series, compounds **39a** and **39b** exhibited potent analgesic and anti-inflammatory activities with no ulcerogenic effect [68]. 
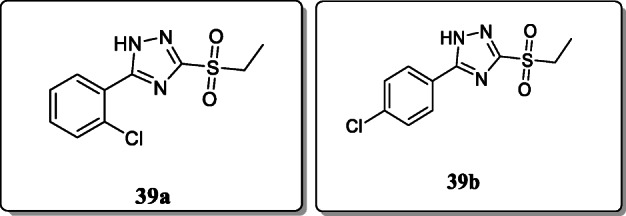


*Kaur et al.* described a novel series of 1,4-diaryl-substituted triazoles was synthesized and evaluated for their COX-2 inhibition. From the result, only compound **40a** displayed excellent COX-2 activity [69]. 
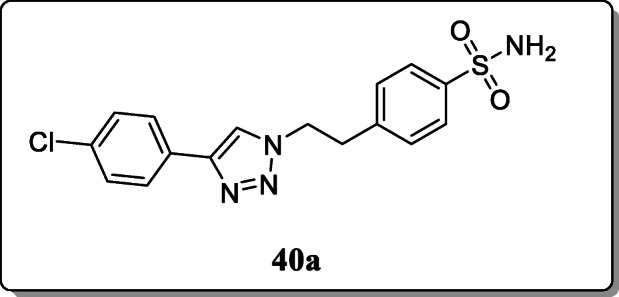


#### Anticancer activity

*Mahanti et al.* reported a series of fused acridine containing 1,2,4-triazole derivatives. And screened their anti-proliferative activity towards several human cell lines including, MCF7 (Breast), A549 (Lung), A375 (Melanoma), and HT-29 (Colon). The IC_50_ value of target compound in range between 0.11 ± 0.02 and 13.8 ± 0.99 μM as compared with the standard range 0.11 ± 0.02 to 0.93 ± 0.056 μM. Result revealed that the compounds **41a**–**41c** exhibited the excellent anticancer activity. SAR investigations of this series revealed that introduction of 4-chloro, 3,4,5-(CH_3_O)_3_, and 4-CF_3_CH_3_ groups at para-position of the phenyl-ring displayed the significant anticancer activity [70]. 
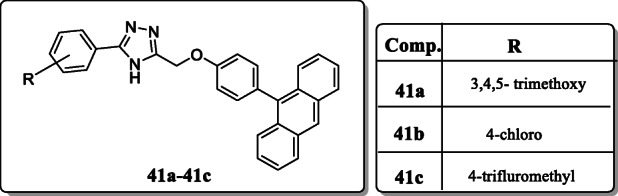


*Al-Wahaibi et al*. reported a novel series of 1,2,4-Triazolyl coumarin derivatives and evaluated their anti-proliferative activity towards human colon cancer cell line (HCT116). Result showed that the compound **42a** exhibited anti-proliferative activity with IC_50_ values 4.363 μM respectively [71]. 
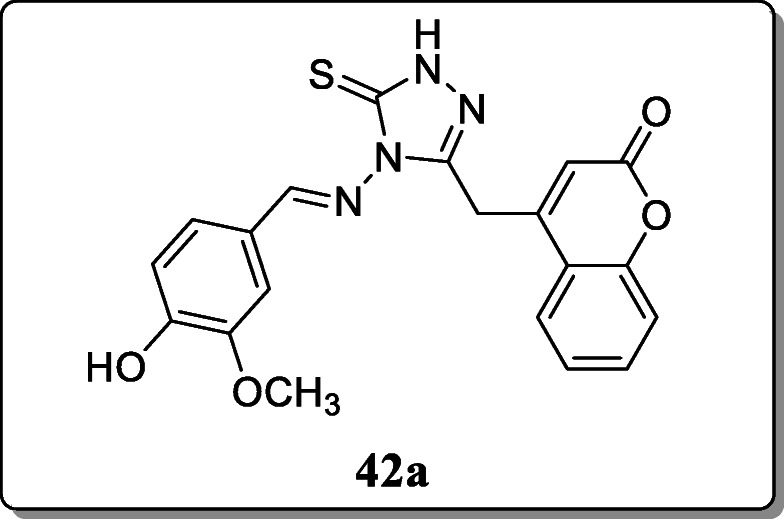


*Ma et al.* synthesized a new series of 1,2,3-triazole-pyrimidine-urea derivatives and evaluated their anti-proliferative activity against selected four different human tumour cell lines including MCF-7, MGC-803, EC-109, and B16-F10. The compounds **43a–43c** exhibited significant growth inhibition against B16-F10 with IC_50_ values of 32 μM, 35 μM, and 42 μM among all the tested compounds [72]. 
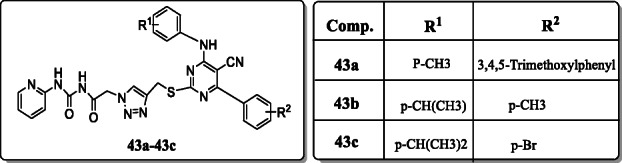


*Ma et al.* reported a novel series of 1,2,3-triazole-pyramidine hybrid derivatives and screened their cytotoxic potential towards several tumour cell lines. Among these synthesized compounds, the compound **44a** exhibited the potent and selective anti-proliferative activity with IC_50_ values in range between 1.42 and 6.52 μM. Particularly, studies revealed that the compound **44a** also inhibit the growth of EC-109 cancer cells via apoptosis-inducing activity and cell cycle arrest at G2/M phase [73]. 
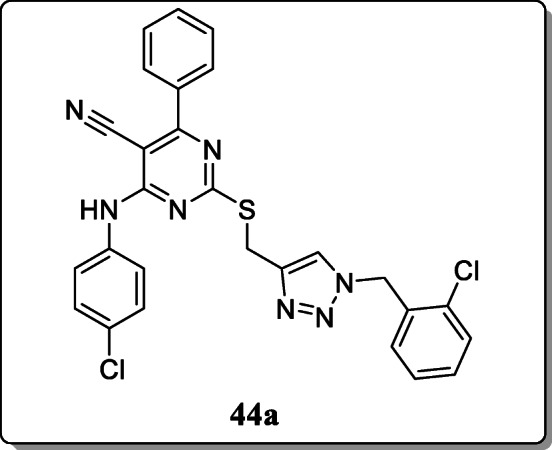


*Duan et al.* synthesized a new series of 1,2,3-triazole-dithiocarbamate hybrids and screened their anticancer activity against four different selected human cancer cell lines including MCF-7, PC-3, MGC-803, and EC-109. Among these, the compounds **45a** and **45b** showed significant wide-spectrum activity. Compound **45a** was found to be most potent towards selected four different human cancer cell lines as compared with 5-fluorouracil [74]. 
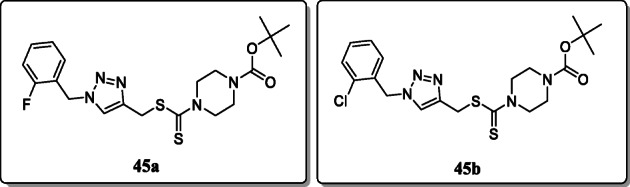


*Aouad et al.* reported a novel series of benzothiazole-piperazine-1,2,3-triazole hybrids and investigated their anti-proliferative activity against different human cancer cell lines. Some hybrid molecules showed significant antiproliferative activity. ADME and clog P analysis method confirmed the biological profile. From the result, compound **46a** exhibited the remarkable antiproliferative activity [75]. 
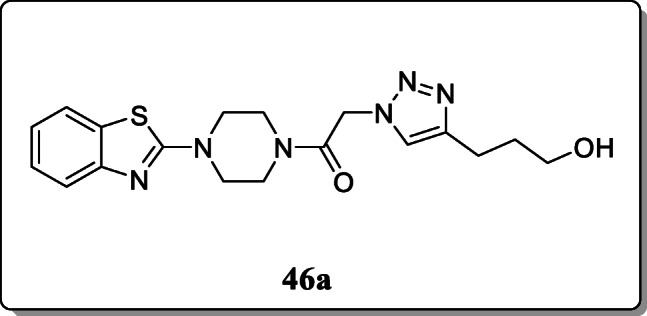


*Ashwin et al.* reported a novel series of 1,2,3-triazole derivatives and screened their anticancer activity against acute myeloid leukemia cell lines. Result revealed that, compound **47a** exhibited the significant anticancer activity with an IC_50_ of 2 μM towards MV4-11 cells [76]. 
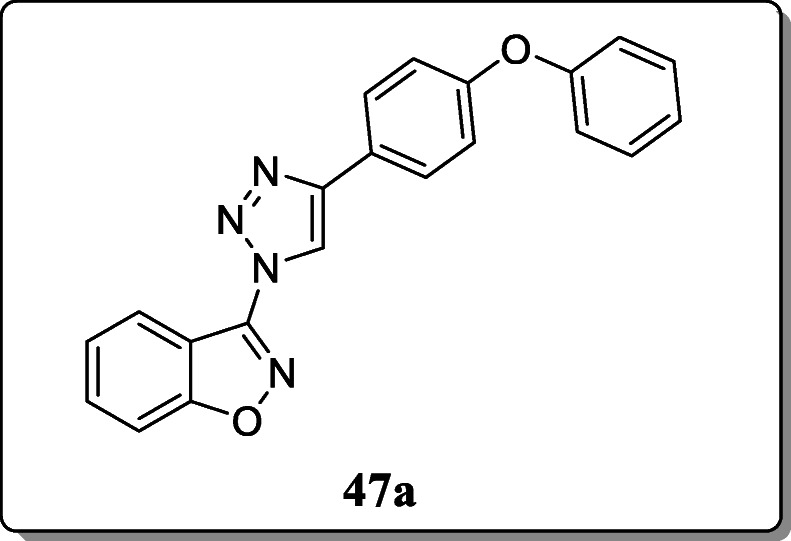


*Dhawan et al.* reported a new series of coumarin-tagged β-lactam triazole hybrids and evaluated for their anticancer activity against different cancer cell lines (MDA-MB-231, MCF7, A549) and one control cell line HEK293. Among the tested series, compounds **48a** and **48b** exhibited excellent activity against MCF-7 cancer cell line with IC_50_ values of 53.55 and 58.62 μM and no cytotoxicity against normal cell line. SAR studies revealed that the presence of nitro and chloro groups at C-3 position of the phenyl ring also enhance their activity against MCF-7 cell line [77]. 
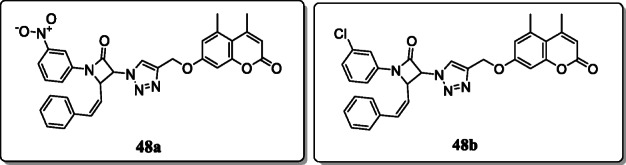


*Saftic et al.* synthesized 8-triazolyl acyclovir derivatives for *in vitro* evaluation of cytostatic activity against Madine Darby canine kidney (MDCK I) cells and different tumour cell lines. From the result, compound **49a** with the shortest alkyl substituent at the triazole ring showed significant inhibitory activity against the CaCo-2 cell line but low cytotoxic effect on normal MDCK I cells [78]. 
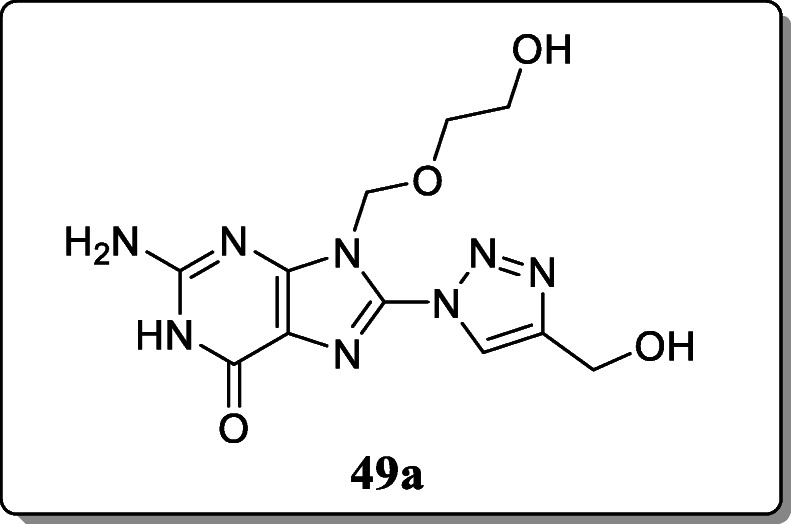


#### Antidiabetic activity

*Saeedi et al*. reported the quinazolinone-1,2,3-triazole hybrid derivatives and screened their *in vitro* α-glucosidase inhibitory activity as leading to an effective antidiabetic agent. All these derivatives displayed excellent antidiabetic activity with IC_50_ values ranging between 181.0 and 474.5 μM and were found to be more potent than reference drug acarbose (IC_50_ = 750.0). Result showed that the compounds **50a** and **50b** where 4-bromobenzyl moiety substituted to the 1,2,3-triazole ring exhibited excellent inhibitory activity with (IC_50_ = 181.0 ± 1.4) and (IC_50_ = 192.3 ± 1.8). Furthermore, *in silico* docking studies showed the binding mode of these analogues on the active site of α-glucosidase [79]. 
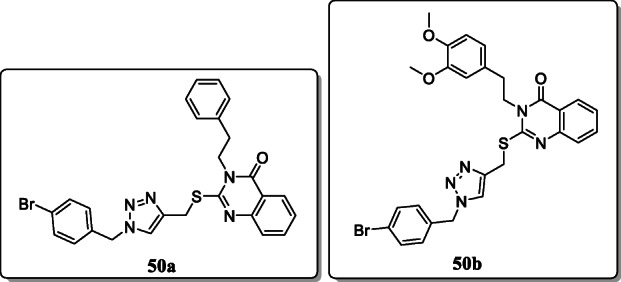


*Avula et al.* synthesized a class of novel 1,2,3-triazole analogues were synthesized and evaluated their α-glucosidase inhibitory activity in ranges between 14.2 and 218.1 μM. Result revealed that the compound **51a** exhibited the most effective antidiabetic activity as compared with the reference drug. And the activity of this compound is 67 times better than the reference due to the presence of the methoxy phenyl group [80]. 
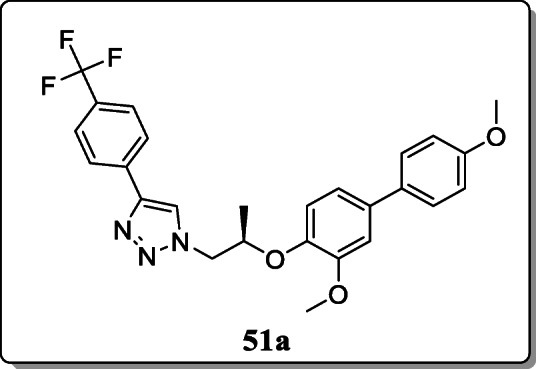


*Wang et al.* synthesized a novel series of triazine-triazole derivatives and evaluated their antidiabetic activity. All these derivatives exhibited the potent antidiabetic activity. Out of all synthesized compounds, compound **52a** showed potent α-glucosidase inhibitory activity [14]. 
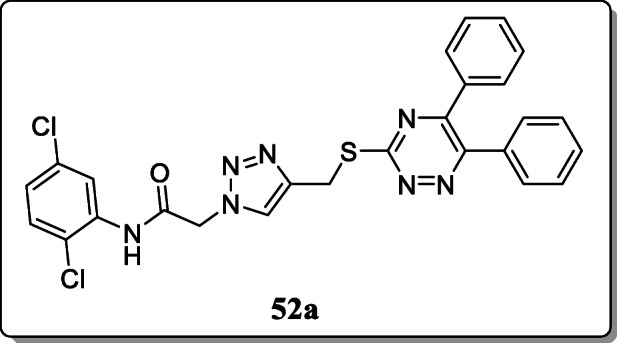


*Chinthala et al.* reported a novel series of chalcone-1,2,3-triazole hybrids and screened their α-glucosidase inhibitor activity. These hybrids exhibited the potential antidiabetic activity. Result showed that the compounds **53a**, **53b**, and **53c** with IC_50_ values of 67.77 μM, 74.94 μM, and 102.10 μM, respectively, exhibited potent α-glucosidase inhibition. Furthermore, the docking studies showed these compounds target the α-glucosidase in range 100.37 to 107.78 [81]. 
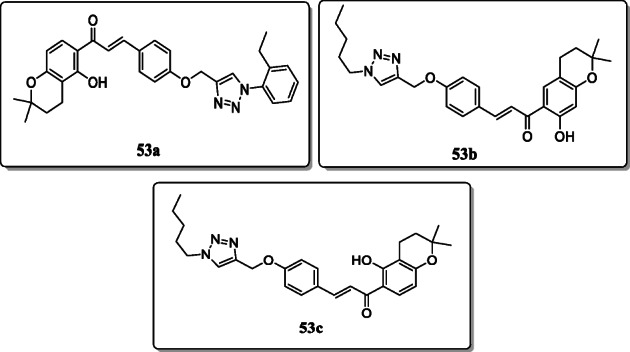


*Gonzaga et al.* synthesized 1-phenyl-1H-2-phenyl-2H-1,2,3-triazol derivatives and screened their α-glucosidase and porcine pancreatic α-amylase activity. All compounds tested at 500 μM, only compound **54a** was found to have the most significant antidiabetic activity with 54 μM as compared with acarbose [82]. 
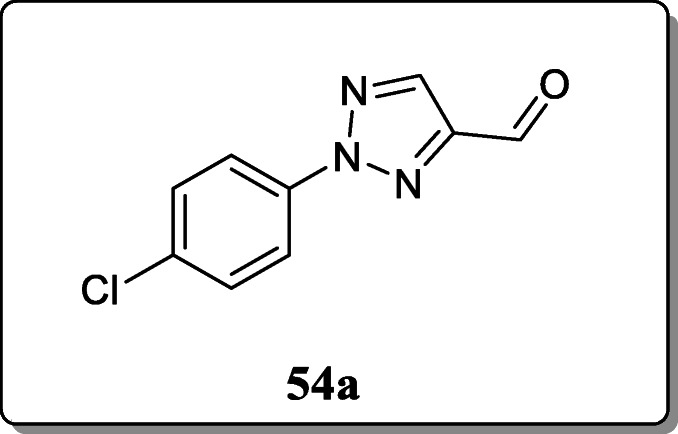


#### Antiviral activity

*Ju et al.* reported a new class of 1,2,3-triazole oseltamivir analogues and screened their antiviral activity against three different strains (H5N1, H5N2, H5N6) in both enzymatic assay and cellular assay. From the result, compound **55a** exhibited the broad-spectrum antiviral activity with IC_50_ value 0.12 μM, 0.049 μM, and 0.16 μM against three different strains [83]. 
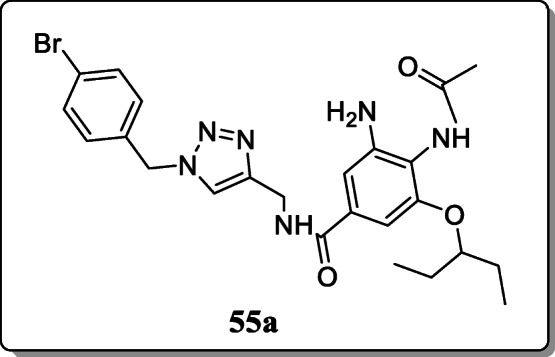


*Jordao et al.* synthesized a novel series of N-amino-1,2,3-triazole compounds and screened their antiviral activity against *Cantagalo virus.* All derivatives were characterized by IR, ^1^H, and ^13^C spectroscopy and elemental analysis. From the result, compound **56a** revealed the excellent antiviral activity [84]. 
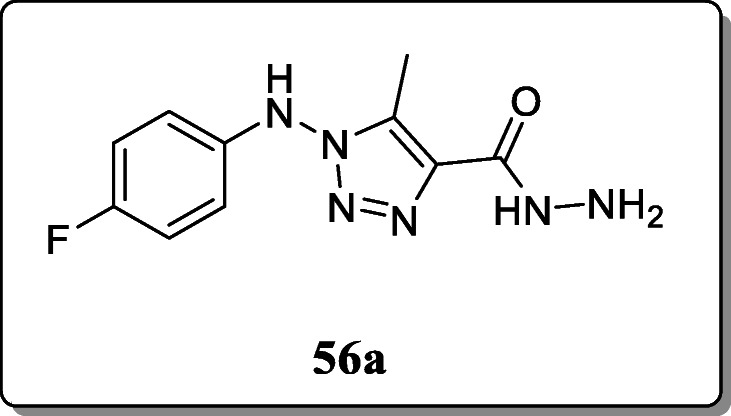


*Kucukguzel et al.* investigated a new series of novel thiourea containing triazole derivatives and tested their anti-HIV activity. Structures of synthesized derivatives were confirmed by elemental and spectral analysis. Result revealed that the compound **57a** exhibited the significant anti-HIV activity towards Coxsackie virus B4. SAR studies revealed that, the presence of the allyl group at N-4 of the 1,2,4-triazole ring and phenyl ring at terminal nitrogen of thioureas enhanced their activity [85]. 
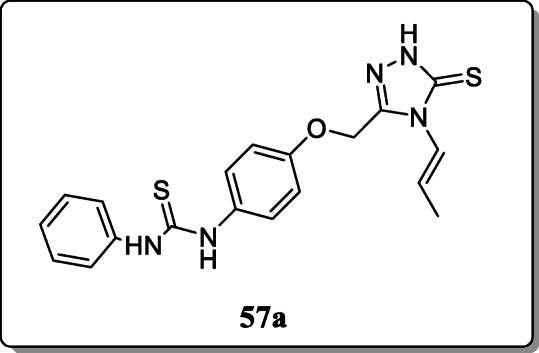


*Wang et al.* reported a novel series of sulfanyl-triazole derivatives as an HIV-1 non-nucleoside reverse transcriptase inhibitor by using high throughput screening. It exhibited significant activities against the selected resistant mutants. From the result, compound **58a** exhibited excellent anti-HIV activity [86].

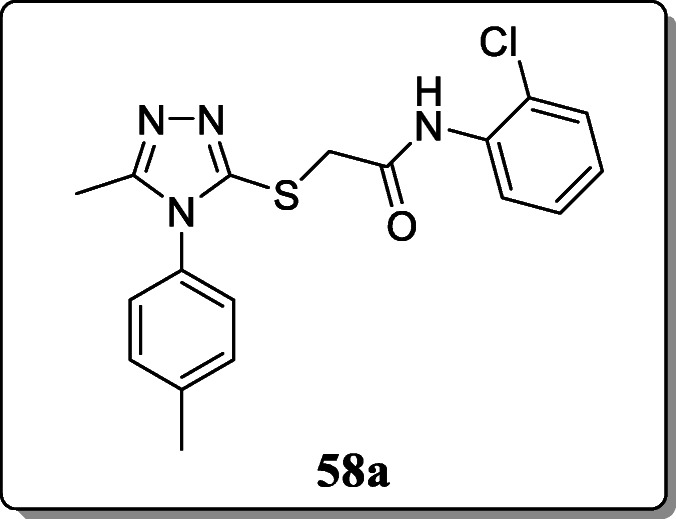



*Karypidou et al.* synthesized a series of fused 1,2,3-triazole derivatives as potential antiviral agent. All the derivatives were screened against some variety of viruses (HIV-1, HIV-2, vaccinia virus, adenovirus-2, and coronavirus) in HEL cells and their inhibitory activity was compared with standard drugs. Among all the tested series, compound **59a** (EC_50_ = 8.95 μM) and **59b** (EC_50_ = 8.90 μM) exhibited the moderate activity against human coronavirus [87].

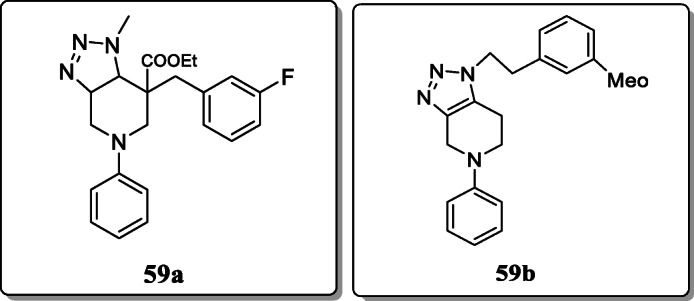



*Cao et al.* synthesized novel triazole derivatives for *in vitro* antiviral activity against EV71 and CVB3 in cell-based assay. All the synthesized derivatives were characterized by various spectroscopic methods including ^1^H NMR, ^13^C NMR, and mass spectroscopy. Among the result, only compound **60a** exhibited remarkable antiviral activities against EV71 and CVB3 virus with the EC_50_ value of 5.3 ± 0.7 and 10.1 ± 3.8 μg/mL as compared with the control ribavirin [88].

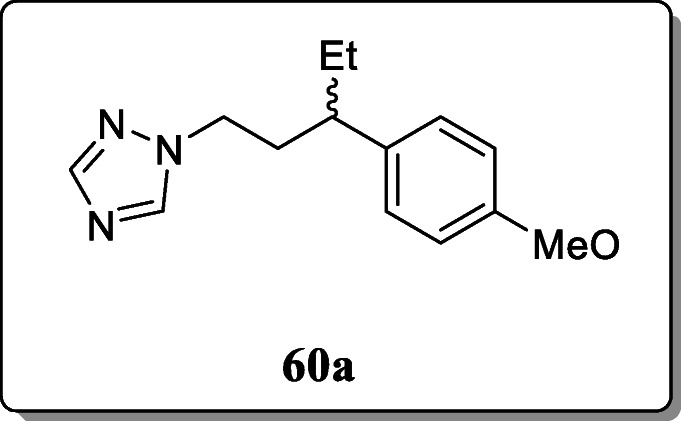



*Mohammed et al.* reported 1,2,3-triazoles as amide bio-isosteres and evaluated for their antiviral activity against H9 and MT4 cells. Result revealed that the 1,4-disubstituted-1,2,3-triazole based derivatives **61a** was found to have significant anti-HIV activity against only H9 cells (IC_50_ = 1.2 μM in H9 cells) and no activity against MT4 cells [89]. 
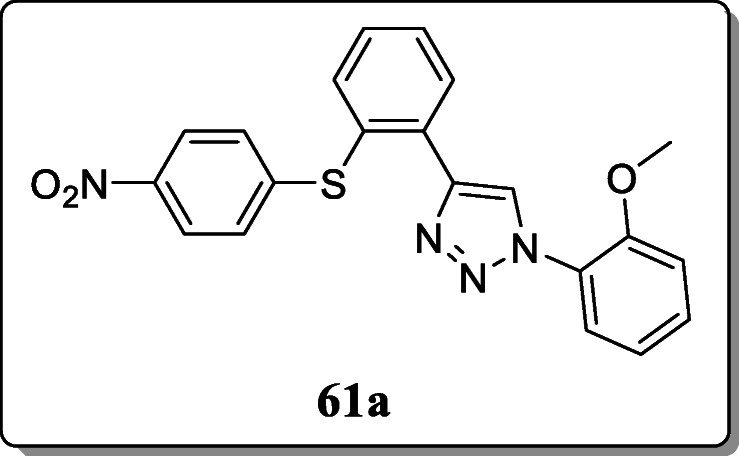


#### Antimalarial activity

*Oramas-Royo et al.* reported and investigated a new series of 1,2,3-triazole-napthaquinone derivatives. Several of these compounds were tested for their *in vitro* antimalarial activity towards *Plasmodium falciparum* strains. From the result, compounds **62a** and **62b** exhibited potent antimalarial activity with IC_50_ values of 0.8 and 1.2 μM. SAR studies revealed that the compound **62a** bearing a fluoro group at C-3 and a methoxy group at C-4 and compound **62b** with an unsubstituted phenyl ring enhanced the antimalarial activity [90]. 
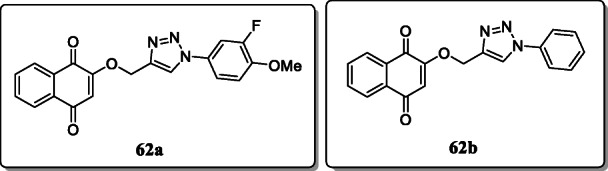


*Thakur et al.* synthesized a novel series of glycosylated 1,2,3-triazolyl-methyl-indoline-2,3-dione derivatives via acid catalyzed reaction and evaluated their anti-plasmodial activity. Among them, compounds **63a** and **63b** exhibited the good activity against resistant strain pfk1 with IC_50_ values 1.61 and 1.93 μM, respectively [91]. 
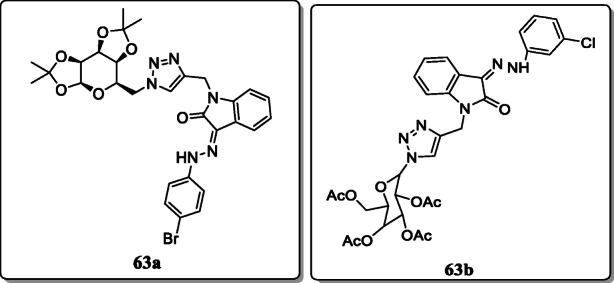


*Thakkar et al.* reported new ten compounds containing 1,2,4-triazole and evaluated their *in vitro* antimalarial activity against *P. falciparum* strain. All these synthesized derivatives were characterized by IR, ^1^H NMR, ^13^C NMR, mass spectroscopy, and elemental analysis. From the result, compounds **64a**, **64b**, and **64c** exhibited the potent antimalarial activity with IC_50_ values 0.282, 0.245, and 0.230 μM as compared with the reference drug chloroquine Pyrimethamine. SAR studies revealed that introduction of 4–OH, 3–NO_2,_ 4–CL in the phenyl group enhance activity [92]. 
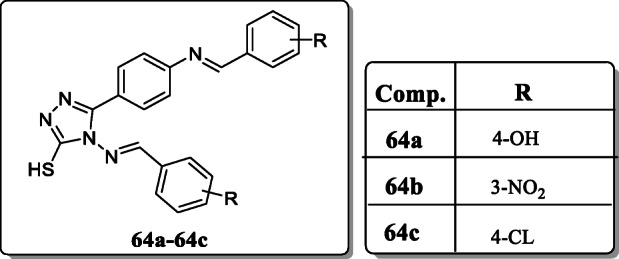


*Joshi et al.* synthesized a novel series of quinoline triazole amide analogues and screened for their antimalarial activity against different strains (CQS D10 and CQR K1). It was concluded that the compounds **65a**, **65b**, and **65c** showed most potent activity towards *P. falciparum* CQS D10 strain with IC_50_ values in the range between 349 and 1247 μM, and these compounds also exhibited similar activity against CQR K1 strain of parasite [93]. 
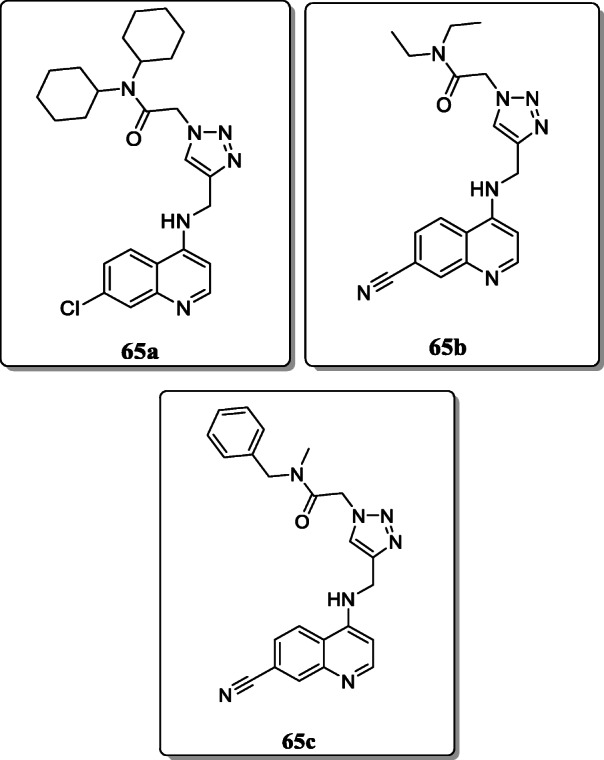


*Guantai et al.* synthesized a new triazole-linked chalcone and dienone hybrids and evaluated *in vitro* antimalarial activity. From the result, compound **66a** was found to have the most significant activity against D10, DD2, and W2 strains of *P. falciparum* as compared with the reference drug chloroquine [94]. 
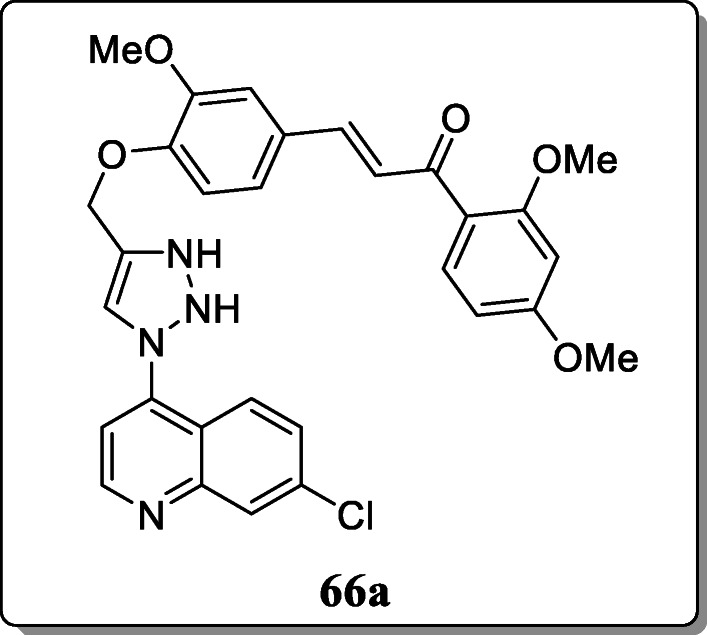


*Tarawneh et al.* synthesized a novel series of isoxazole and triazole derivatives and evaluated for their anti-infective agent. All the compounds were screened against *P. falciparum* D6 and W2 strains. From the result, the only compound **67a** exhibited the most potent activity with IC_50_ values of 0.70 and 0.59 μM against D6 and W2 strains [95]. 
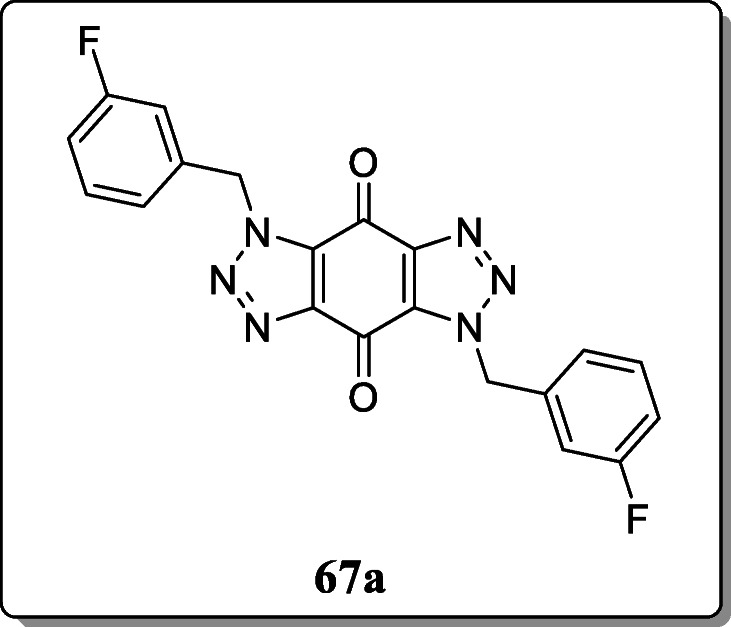


### Miscellaneous activities

In spite of all these activities, triazoles are also active as antihypertensive agent **68a**, neuroprotective agents **68b** and **68c**, and diuretic **68d** (Table 3). Triazole nucleus was found to possess significant atypical behaviour and good potency to block 5-HT receptors and good ability of selective antagonists towards the human vasopressin V_1A_ receptor [96–99]. 
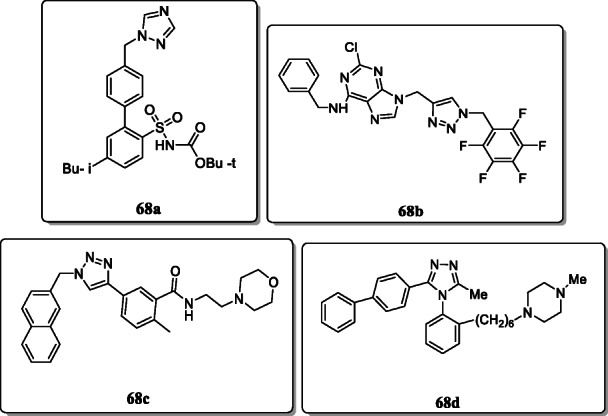

**Table 3:**
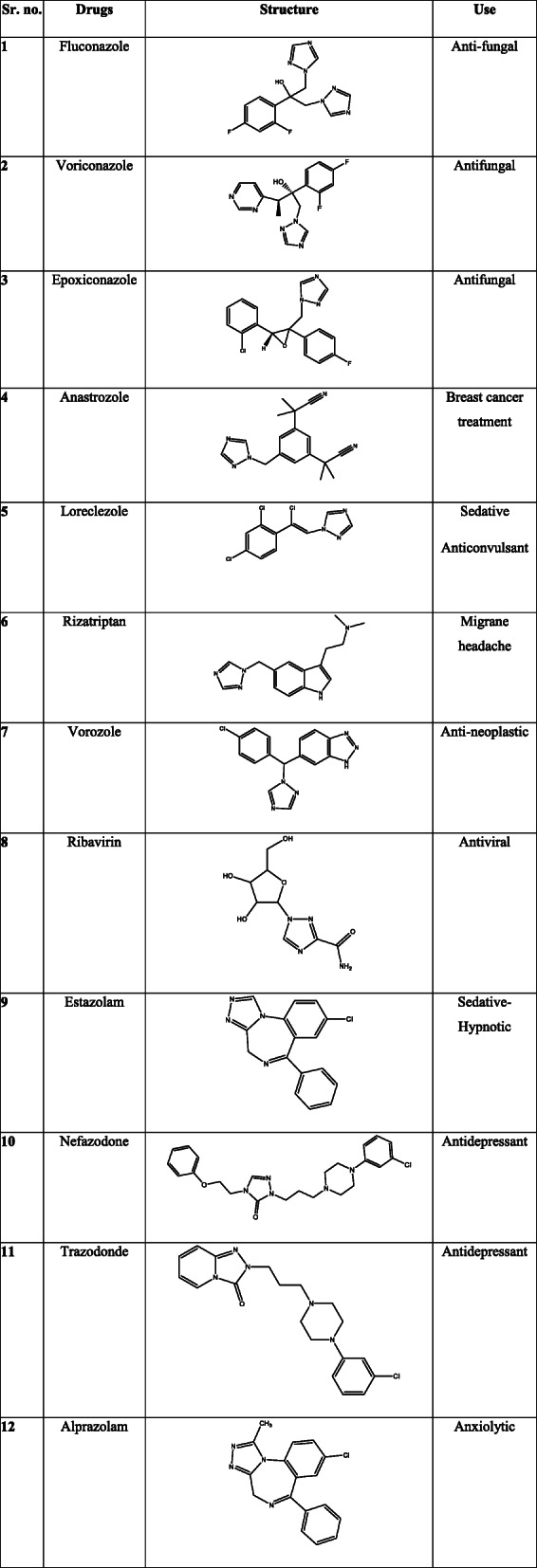
Some triazole nucleus-containing drugs in market and their clinical use [94–105]

## Conclusion

This review article highlights research work of many researchers reported in literature for different pharmacological activities on triazole compounds. Triazole has unique moiety that is responsible for various biological activities. The importance of triazole moiety can be magnified by carrying out further studies on its possible substitution and thus to synthesize better agents that can have strong future commitments. This review has presented comprehensive details of triazole analogues, potent compounds reported for particular pharmacological activity and the method or technique involved in evaluation process. More investigations must be carried out to evaluate more activities of triazole for many diseases whose treatment are difficult in the medical sciences.

## Data Availability

All the information in the manuscript has been referred from the included references and is available upon request from the corresponding author.

## Abbreviations

IC_50_: Half maximal inhibitory concentration
SAR: Structure–activity relationship
EC_50_: Median effective concentration required to induce a 50% effect
COX: Cyclooxygenase
MBC: Minimum bactericidal concentration
MIC: Minimum inhibitory concentration
PEG: Polyethylene glycol
PI: Plaque index
IR: Infrared
NMR: Nuclear magnetic resonance
